# Left ventricular remodeling response to SGLT2 inhibitors in heart failure: an updated meta-analysis of randomized controlled studies

**DOI:** 10.1186/s12933-023-01970-w

**Published:** 2023-09-02

**Authors:** Erberto Carluccio, Paolo Biagioli, Gianpaolo Reboldi, Anna Mengoni, Rosanna Lauciello, Cinzia Zuchi, Sandra D’Addario, Giuliana Bardelli, Giuseppe Ambrosio

**Affiliations:** 1 Cardiology and Cardiovascular Pathophysiology, Santa Maria della Misericordia Hospital, University of Perugia, Perugia, Italy; 2 Department of Medicine and Surgery, University of Perugia, Perugia, Italy; 3 CERICLET- Centro Ricerca Clinica e Traslazionale, University of Perugia, Perugia, Italy

**Keywords:** SGLT2i, Cardiac magnetic resonance imaging, Echocardiography, Cardiac remodelling, HFrEF, HFpEF

## Abstract

**Background:**

Randomized controlled trials (RCTs) reported contrasting results about reverse left ventricular remodeling (LVR) after sodium-glucose co-transporter-2 inhibitors (SGLT2i) therapy in patients with heart failure (HF).

**Methods and results:**

We performed a metanalysis of RCTs of SGLT2i administration in HF outpatients published until June 2022 searching four electronic databases. The protocol has been published in PROSPERO. Primary LVR outcome was change in absolute LV end-diastolic (LVEDV) and end-systolic volume (LVESV) from baseline to study endpoint. Secondary outcomes included changes in LVEDV and LVESV indexed to body surface area, LV Mass index (LVMi), LV ejection fraction (LVEF), and N-terminal pro-B-type natriuretic peptide (NTproBNP). Mean differences (MDs) with 95% CIs were pooled. A total of 9 RCTs (1385 patients) were analyzed. All of them reported data on LVEF. Six trials reported data on LVESV and LVEDV (n = 951); LVMi was available in 640. SGLT2i treatment significantly reduced LVEDV [MD= -10.59 ml (-17.27; -3.91), P = 0.0019], LVESV [MD= -8.80 ml (-16.91; -0.694), P = 0.0334], and LVMI [MD= -5.34 gr/m2 (-9.76; -0.922), P = 0.0178], while LVEF significantly increased [MD = + 1.98% (0.67; 0.306), P = 0.0031]. By subgroup analysis, the beneficial effects of SGLT2i on LVEF did not differ by imaging method used, time to follow-up re-evaluation, or HF phenotype. Reduction in LV volumes tended to be greater in HF with reduced EF (HFrEF) than in those with preserved EF (HFpEF), while the opposite was observed for LVMi.

**Conclusions:**

Treatment with SGLT2i significantly reversed cardiac volumes, improving LV systolic function and LV mass, particularly in HFrEF patients.

**Supplementary Information:**

The online version contains supplementary material available at 10.1186/s12933-023-01970-w.

## Introduction

Initially introduced as a major therapeutic advance in the field of type-2 diabetes mellitus (T2DM) to aid glycemic control, sodium-glucose co-transporter-2 inhibitor (SGLT2i) drugs have also demonstrated to strongly reduce the incidence of a composite endpoint of cardiovascular death/HF re-hospitalizations both in heart failure (HF) patients with reduced ejection fraction (HFrEF) [1, 2] and, more recently, in HF patients with preserved EF (HFpEF) [3, 4]. Interestingly, in all these studies the beneficial effects of SGLT2i on outcome were observed in patients regardless of T2DM status [1–4], thus making these drugs a new major weapon in the arsenal of available medications used to fight HF.

How SGLT2-inhibitors exert such striking clinical benefits in HF is not completely known, and their beneficial effects cannot be simply explained by their actions on glycemic control, or as osmotic diuretics [5]. Recently, randomized controlled trials (RCT), enrolling patients with T2DM with or without HF, have explored the capability of these drugs to favorably impact left ventricular remodeling (LVR) indices (LV volumes, myocardial mass, and LV systolic function), either assessed by echocardiography or cardiovascular magnetic resonance (CMR), providing contrasting results [6–18]. Therefore, these aspects remain controversial.

We performed an updated metanalysis of RCTs in patients with HF to evaluate the effects of chronic administration of SGLT2-inhibitors on LV volumes, mass, ejection fraction (EF), and N-terminal pro-B-type natriuretic peptide (NTproBNP).

## Methods

### Data source

This meta-analysis was performed in accordance with the Preferred Reporting Items for Systematic Reviews and Meta-Analyses (PRISMA) guidelines. The protocol has been published in the PROSPERO International prospective register of systematic reviews (CRD42023264057). Using the search algorithm detailed in the Supplementary Material Data file, four electronic databases [PUBMED, Web Of Science (WOS), Cochrane Central Register of Controlled Trials, OVID MEDLINE] were searched for RCTs published until June 2022 without any restriction regarding study duration and sample size. Reference lists of all identified studies were also manually searched for further relevant investigations. Reviews, meta-analyses, case reports, summary articles, letters without data, conference abstracts, editorials, posters, nonhuman studies, and non-English articles were excluded.

### Study Selection and eligibility criteria

Two investigators (P.B. and A.M.) independently reviewed the titles and abstracts of all citations retrieved, selected eligible studies, and extracted valuable data from downloaded articles, according to the inclusion and exclusion criteria. Discrepancies were resolved by a third author. Studies were eligible for the analysis in the following cases: (1) they were full-text and relevant data could be acquired; (2) they had to provide baseline and follow-up data for LV end-diastolic (LVEDV) and end-systolic volumes (LVESV), or LV ejection fraction, either assessed by CMR or echocardiography; (3) randomized comparison between SGLT2-inhibitors and placebo or active control.

At least two studies reporting outcome variables were required to be eligible for the analysis. Studies that did not provide enough data to analyze the effect on LV remodeling parameters were excluded, as were studies enrolling patients with type-1 diabetes mellitus (T1DM), or patients aged less than 18 years.

### Definition of outcomes

Our primary efficacy outcome was the difference in the mean change (baseline versus follow-up evaluation) between the treatment group and control group in LVEF, LVEDV, and LVESV. Secondary endpoints were change in LV volumes indexed by body surface area (BSA), LV mass index (LVMi), and NT-proBNP.

### Data extraction and quality assessment

The above reported investigators independently extracted relevant outcome data and any disagreement was resolved by consensus in discussion with all authors. The following data were extracted: first author’s name, study’s acronym, publication year, study location, sample size, patient characteristics (sex, age, previous medication, estimated glomerular filtration rate [eGFR]), setting [HFrEF, HFpEF, T2DM], type of SGLT2-inhibitor, time to follow-up re-evaluation, and method of measurement (echocardiography, CMR). The methodological quality of the included studies was evaluated using the Cochrane Risk of Bias Tool [19] across the domains of sequence generation, allocation concealment, blinding, incomplete outcome data, and selective reporting.

### Statistical analysis

We used mean±standard deviation (SD) change from baseline to calculate the pooled effects. If only the standard error or 95% confidence intervals (CIs) were reported, SD was calculated as described by Altman and Bland [20] and Cochrane Handbook, respectively [19]. If only median values with interquartile range were reported, means and SDs were estimated using the Box-Cox method [21]. For studies reporting only baseline and final measurements separately, but not change-from-baseline mean (SD), a correlation coefficient was calculated according to other included studies which provided mean (SD) for baseline, final, and change values. A conservative estimate (minimum correlation) was used to impute the missing SD of mean changes [22].

The pooled effect size was summarized as mean difference (MD) with corresponding 95% CIs according to the inverse-variance method. A random-effects model was applied to compute the effect size with 95% CIs. Heterogeneity between studies was assessed using the Q statistic, and its extent was calculated by the I [2] test, with an I [2] value > 50% indicating high heterogeneity. Intention-to-treat analysis was used wherever possible.

A predefined subgroup analysis was performed by stratifying according to imaging methods used (echocardiography, CMR), time to follow-up re-assessment (</≥6 months), and HF phenotype. Since the reporting studies used different imaging methods, sensitivity analysis was performed to evaluate the robustness of the effects by using standardized mean difference (SMD) for LV volumes data. Publication bias was tested visually using the funnel plot and quantitatively using the Begg adjusted-rank correlation test [23]. Meta-analyses were performed using R software (version 4.2.1; 2022, The R Foundation for Statistical Computing; https://www.r-project.org) and STATA (version 17, StataCorp, Lakeway Drive, College Station, Texas, USA).

## Results

### Search results and baseline characteristics

**Fig. 1:**
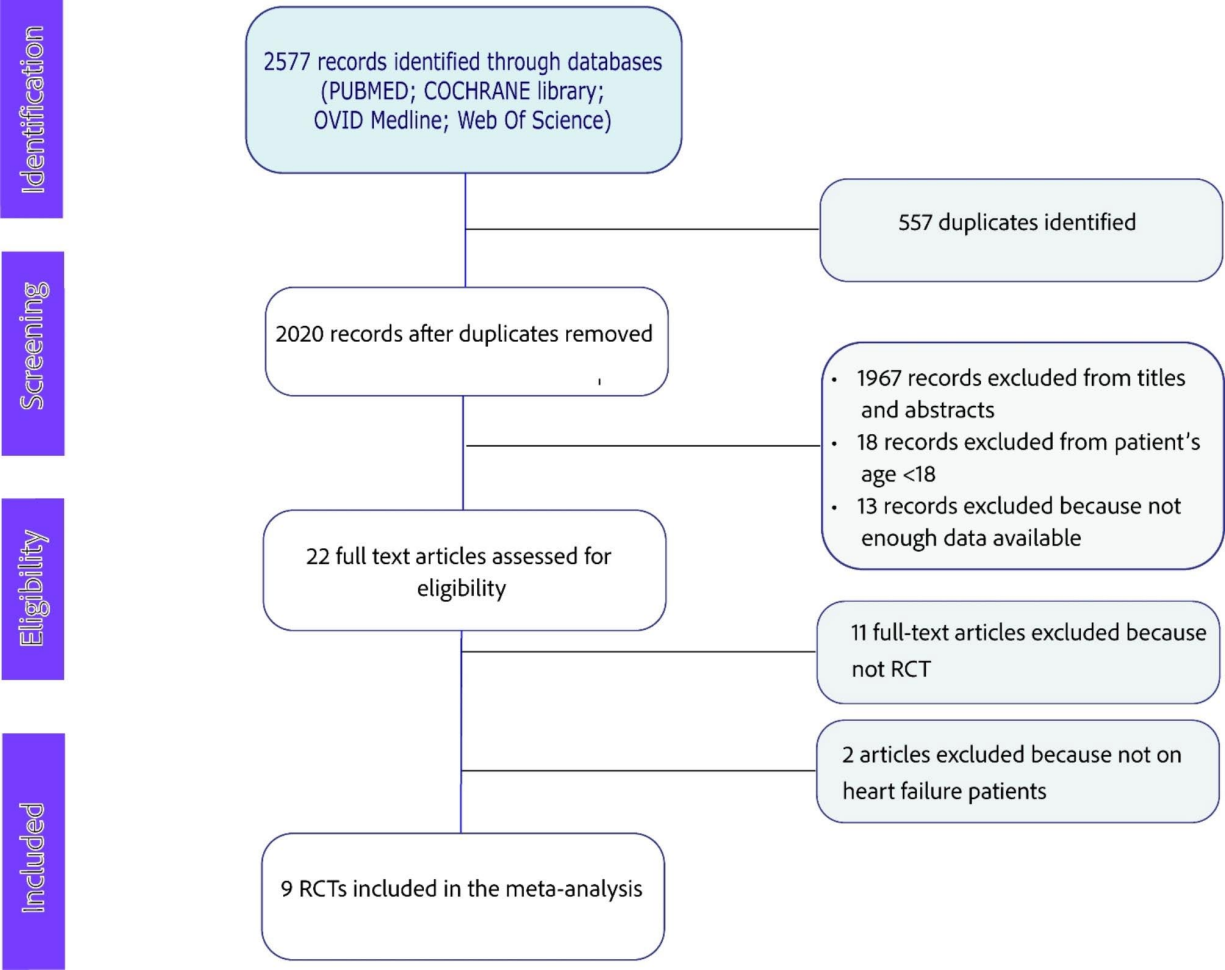
Flow diagram showing detailed study selection process

A total of 9 studies met the inclusion criteria and were analyzed in this metanalysis (detailed flowchart in Figure-1). Baseline characteristics of the included studies are summarized in Table 1. The year of publication ranged from 2020 to 2022. A total of 1385 patients were included, 685 of whom had been assigned to SGLT2i (empagliflozin in 3 studies, dapagliflozin in 1 study, canagliflozin in 3 studies, Ipragliflozin in 1 study, and Luseogliflozin in 1 study; Table-1). The mean patient age ranged from 56.2 to 73.2 years, and 89.7% of subjects were male. A total of 987 patients (in 7 studies) had T2DM with HFrEF, while three studies (398 patients) reported data on T2DM with HFpEF. Of the 9 included studies, 8 reported data on concomitant treatment with renin-angiotensin-system (RAS) inhibitors [angiotensin-converting enzyme inhibitors (ACEi), angiotensin-receptor blockers (ARBs), angiotensin-receptor-neprilysin inhibitors (ARNI)], and beta-blockers (Table-1). Use of RAS-inhibitors (89.8% versus 90.4%, *P* = 0.7086) and beta-blockers (94% versus 94.1%, *P* = 0.937) did not significantly differ between patients assigned to SGLT2-inhibitors or controls. The follow-up duration ranged from 3 to 12 months (median xxxxxx). LV imaging was performed with CMR in 3 studies [10, 14, 15], and the remaining with echocardiography [6, 8, 9, 12, 16, 18].

**Table 1:** Characteristics of Included Studies and Patients of the Meta-Analysis

Trial, First Author, year	Active treatment	Imaging Modality	Setting	N° Patients	Age	Males (%)	FU (mo)	LV Remodeling Indices	Beta-blockers, (%)	ACEi/ARBs/ARNI, (%)
*SUGAR-DM-HF trial* *Lee Matthew MY, 2021*	Empagliflozin	CMR	HFrEF	92	69±11	73	9	EDV, EDVi, ESV, ESVi, EF, LVMi,	91	100
*EMPA-TROPISM*, *Santos-Gallego CG et al., 2021*	Empagliflozin	CMR	HFrEF	80	62±12	64	6	EDV, ESV, EF, LVM	88	85
*EMPIRE-HF*, *Omar Massar et al., 2021*	Canagliflozin	Echo	HFrEF	179	64±11	85	3	EDV, EDVi, ESV, ESVi, EF, LVMi,	95	100
*The REFORM Trial*, *Singh JSS et al., 2020*	Dapagliflozin	CMR	HFrEF	56	67±7	66	12	EDV, EDVi, ESV, ESVi, EF, LVMi	82	89
*CANA-HF*, *Carbone S, 2020*	Canagliflozin	Echo	HFrEF	36	56±7	78	3	EDVi, ESVi, EF, NTproBNP	94	86
*EMMY Trial* *Von Lewinski D et al., 2022*	Empagliflozin	Echo	HFrEF	476	58±9	82	6.5	EDV, EDVi ESV, ESVi, EF, NTproBNP	96	98
*EXCEED Trial*, *Akasaka H et al., 2020*	Ipragliflozin	Echo	HFpEF	68	71±8	60	6	EDV, ESV, EF, LVMi	NA	NA
*CANDLE Trial*, *Tanaka A. et al., 2020*	Canagliflozin	Echo	HFrEF, HFpEF	233	69±10	75	6	EF, NTproBNP	70	76
*MUSCAT-HF*, *Ejiri K. et al., 2020*	Luseogliflozin	Echo	HFpEF	165	73±7	62	3	EF, LVMi, NTproBNP	61	59

### Risk of bias assessment

The risk of bias assessment is reported in detail in the Supplementary material data (Supplemental Tables-S1 and Figure-S1). The overall risk of bias was low in most RCTs.

### Left ventricular end-diastolic volume and volume-index

**Fig. 2:**
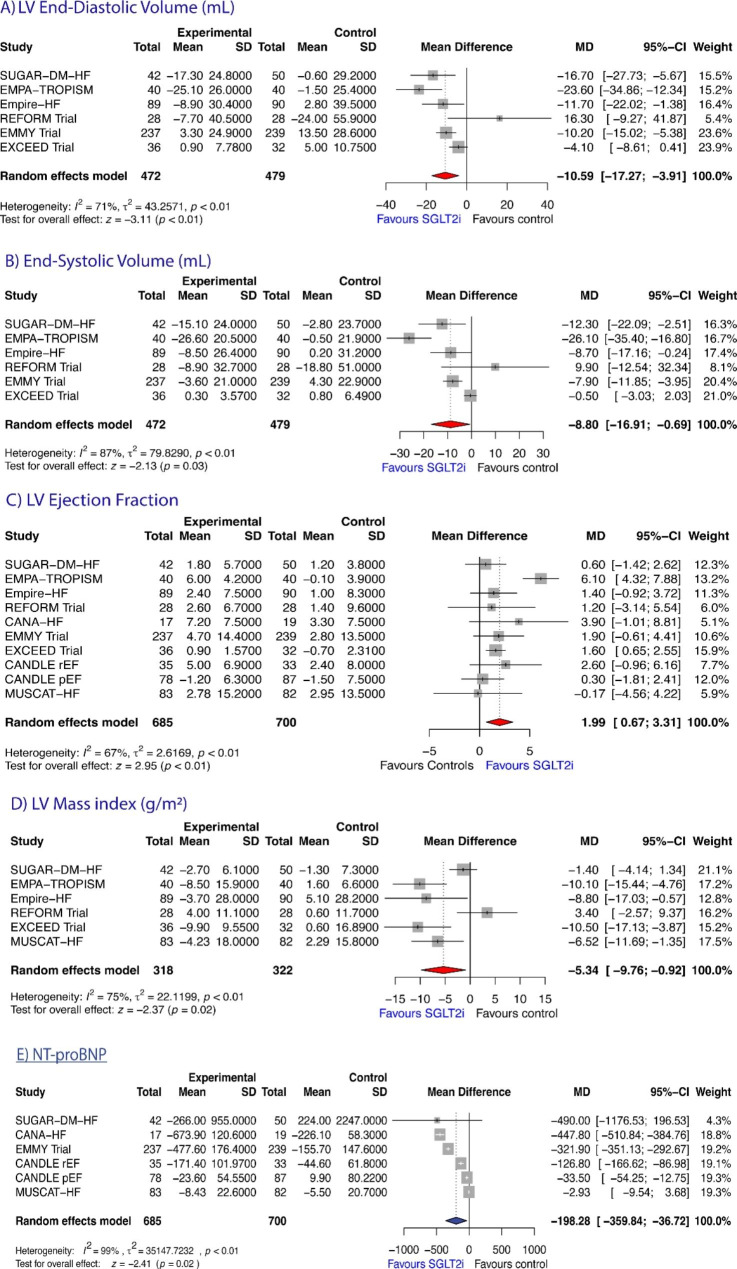
Forest plots showing the effects of SGLT2 inhibitors on (**A**) LV end-diastolic volume; (**B**) LV end-systolic volume; (**C**) LV Ejection Fraction; (**D**) LV Mass Index; and (**E**) NT-proBNP.

**Fig. 3:**
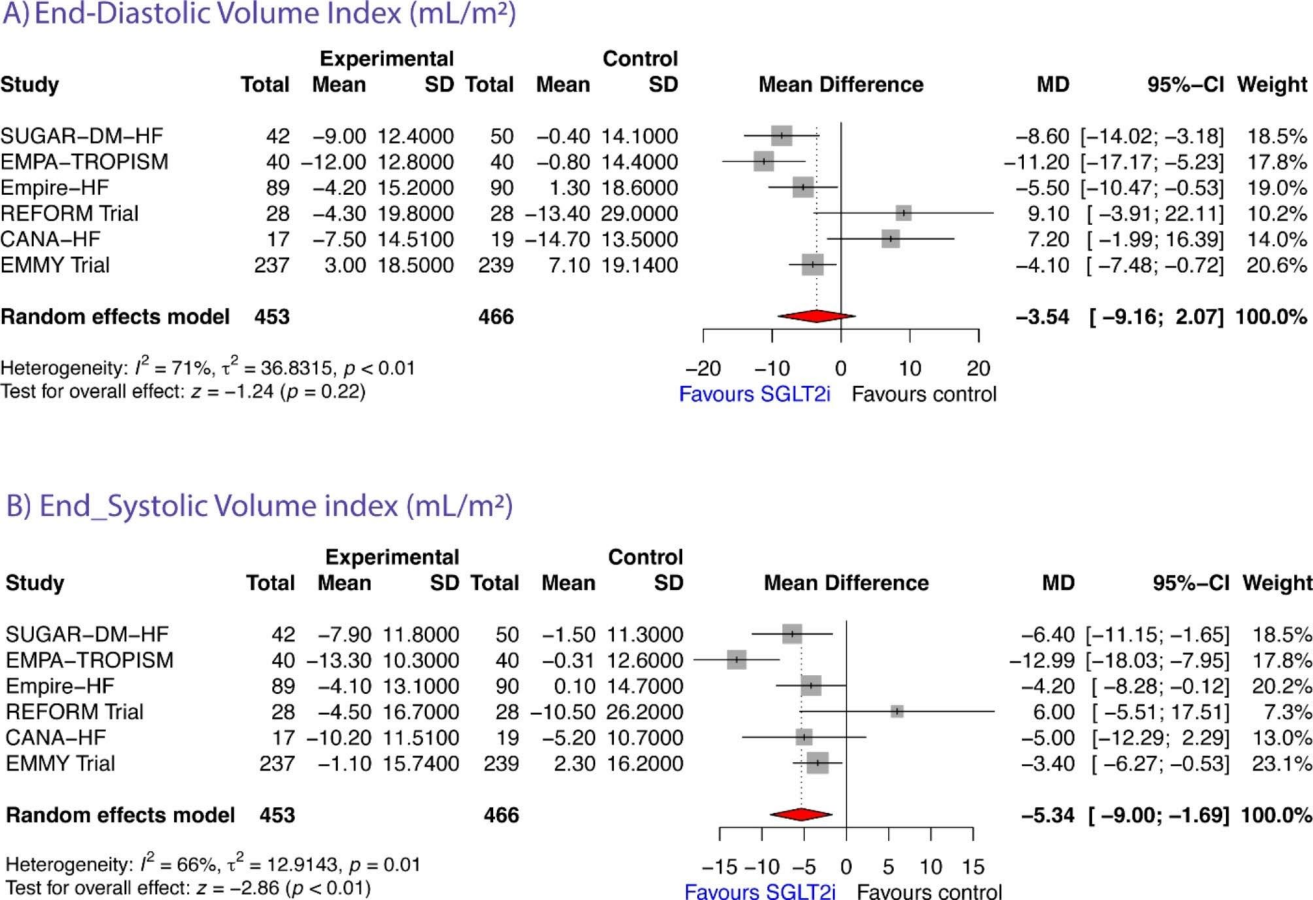
Forrest plot showing changes in end-diastolic volume index (**A**) and end-systolic volume index (**B**) from baseline to study endpoint in randomized controlled trials of heart failure patients treated with sodium glucose transporter-2 inhibitor therapy versus controls

Five studies reported data on both LVEDV and LVEDV-index [10, 12, 14, 15, 18], 1 study reported data only on LVEDV [6], and 1 study reported data only on LVEDV-index [8]. Therefore, a total of 6 studies reported data on LVEDV [6, 10, 12, 14, 15, 18] (n = 951). The pooled data from these studies showed that, compared to controls, LVEDV significantly decreased after SGLT2i by -10.59 mL (95% CI: -17.27 to -3.91; z = -3.11, *P* = 0.0019, Figure-2A). This effect was not observed when LVEDV was indexed by BSA [MD = 3.54 (95% CI: -9.16 to 2.07; z = -1.24, *P* = 0.2163, Figure-3A). According to the Q-test, (***LVEDV***: Q test = 17.10, *P* = 0.0043, tau² = 43.26, I² = 80.8%; ***LVEDV-index***: Q = 17.44, *P* = 0.0037, tau² = 36.83, I² = 71.3%) the true outcome appears to be heterogeneous. Subgroup analyses showed no significant difference in the mean reduction of LVEDV measured by either imaging method (CMR versus echocardiographic studies; Q = 0.07, P = 0.7916; Supplemental Figure-S2, top), or time to follow-up re-evaluation (Q = 0.04, P = 0.8329; Supplemental Figure-S2, bottom), while the effect on LVEDV was more evident for HFrEF than for HFpEF patients (Q = 4.65, P = 0.0310; Supplemental Figure-S2, center). Neither the rank correlation nor the regression test indicated any funnel plot asymmetry (*P* = 0.4655 and *P* = 0.8121, respectively, Supplemental Table-S1).

### Left ventricular end-systolic volume and volume-index

A total of 6 studies reported data on LVESV [6, 10, 12, 14, 15, 18] (n = 951). Of them, five studies reported data on both LVESV and LVESV-index [10, 12, 14, 15, 18], 1 study reported data only on LVESV [6], and 1 study reported data only on LVESV-index [8]. The observed MDs ranged from − 0.50 to -26.10. The estimated average MD based on the random-effects model was − 8.80 mL (95% CI: -16.91 to -0.69); Fig. 2B); therefore, the average outcome differed significantly from “zero” hypothesis (z = -2.13, p = 0.0334) in SGLT2i group compared to controls. This effect remained significant when LVESV was indexed by BSA [MD = -5.34 mL (95% CI: -9.00 to -1.69; z = -2.86, *P* = 0.0042; Figure-3B). According to the Q-test, (***LVESV***: Q test = 38.11, *P* < 0.0001, tau² = 79.83, I² = 86.9%; ***LVESV-index***: Q = 14.79, *P* = 0.0113, tau² = 12.91, I² = 66.2%), once again the true outcomes appear to be heterogeneous.

Subgroup analyses showed no significant difference in the mean reduction of LVESV measured by either imaging method (Q = 0.45, P = 0.5020; Supplemental Figure-S3, top) and time to follow-up re-evaluation (Q = 0.01, P = 0.9994; Supplemental Figure-S3, bottom); however, the effect of SGLT2i on LVESV was more evident for HFrEF than for HFpEF patients (Q = 5.32, P = 0.0211; Supplemental Figure-S2, center). There was no funnel plot asymmetry by both the rank correlation and the regression test (*P* = 0.3481 and *P* = 0.6673, respectively, Supplemental Table-S1), suggesting no evidence of publication bias.

### Effects of SGLT2i on LVEF

**Fig. 4:**
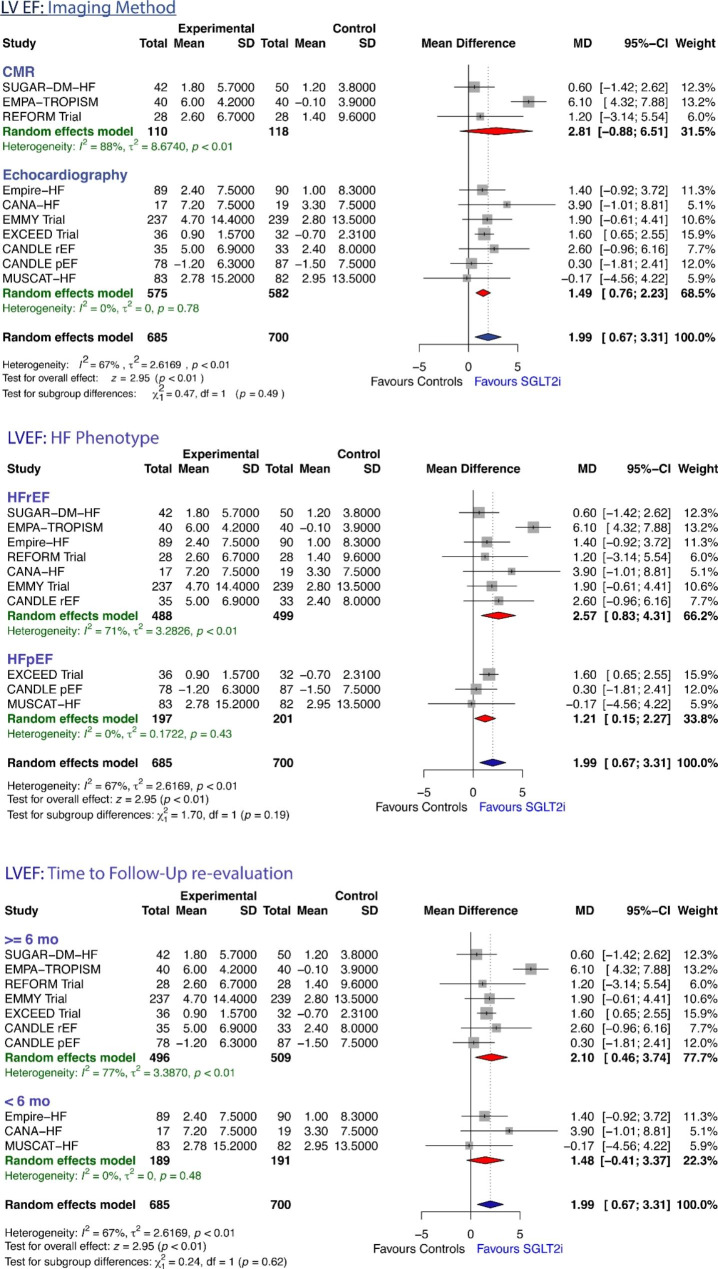
Forrest plot showing changes in LV Ejection Fraction from baseline to study endpoint in randomized controlled trials of heart failure patients treated with sodium glucose transporter-2 inhibitor therapy versus controls, according to pre-specified subgroups

LVEF data were available for all the 9 studies [6, 8–10, 12, 14–16, 18]. The pooled data from these studies showed that, based on the random-effects model, LVEF significantly increased with SGLT2i therapy compared with controls (MD + 1.99%, 95%CI: 0.67 to 3.31, z = 2.95, P = 0.0031; Fig. 1C). The effects on LVEF were not homogeneous across studies (Q test = 27.51, *P* = 0.0011, tau² = 2.62, I² = 67.3%). No significant difference in the mean increase in LVEF between patients treated with SGLT2i and controls were seen according to subgroup analyses based on imaging method, HF phenotype (HFrEF vs. HFpEF), and time to follow-up re-evaluation (Figure-4). Neither the rank correlation nor the regression test indicated any funnel plot asymmetry (P = 0.3481 and P = 0.6673, respectively, Supplemental Table-S1), suggesting no evidence of publication bias.

### Effects of SGLT2i on LV mass index

Information on LVM index was available in 6 studies [6, 9, 10, 12, 14, 15] (n = 640). The pooled analysis of these studies according to a random effect model showed that treatment with SGLT2i was associated with a significant decrease in LV mass index (MD = -5.34 g, 95% CI -9.76 to -0.92, z=-2.37, P = 0.0178, Figure-3A), without any significant differences among subgroups analyzed (Supplemental Figure-S4). Even for LVM index heterogeneity of the effect SGLT2i was seen across studies (Q test = 20.28, *P* = 0.0011, tau² = 22.12, I² = 75.3%).

### Effects of SGLT2i on NT-proBNP values

A total of 6 studies reported data on NT-proBNP values [8–10, 16, 18] (n = 1385). The observed MD ranged from − 2.93 to -490.0. The estimated average MD based on the random-effects model was − 198.28 pg/mL (95% CI: -359.84 to -36.71; Figure-3B); therefore, the average outcome differed significantly from zero (z = -2.41, *P* = 0.0162) in SGLT2i group compared to controls. According to the Q-test, (Q test = 639.23, *P* < 0.0001, tau² = 35147.7, I² = 99.2%) the true outcome appears highly heterogeneous among the studies. There was no evidence of publication bias as indicated by a non-significant rank correlation and regression test (*P* = 0.4524 and *P* = 0.3161, respectively, Supplemental Table-S1).

### Sensitivity analysis

To evaluate the robustness of the effect, sensitivity analyses was performed by using standardized mean difference (SMD) for LV volumes, LVEF, and LVM-index. Analyses showed that the effect size remained of similar size and direction, suggesting that the trends identified in the main analysis remained unchanged (***LVEDV***, SMD: -0.399 mL, *95%CI*: -0.67 to -0.128, P = 0.0039; I [2]: 62.2%; ***LVESV***, SMD: -0.38 mL, *95%CI*: -0.73 to -0.028, P = 0.0341; I [2]: 73.6%; ***LVEF***, SMD: +0.34 mL, *95%CI*: 0.072 to 0.62, P = 0.0134; I [2]: 75.1%; ***LVM-index***, SMD: -0.37 mL, *95%CI*: -0.65 to -0.088, P = 0.0102; I [2]: 61.9%) (Supplemental Figure-S5).

## Discussion

The present meta-analysis provides updated data on the effects of SGLT2i on LV remodeling indices (LV volumes, mass, EF) based on all available studies involving diabetic patients with HF. The pooled results of this analysis show significant improvement in LV volumes and LVEF, as well as a reduction in LVM index and NTproBNP, following SGLT2i treatment compared with controls. The improvement in LVR indices was evident regardless of the imaging method used (echocardiography or CMR), time to LVR reassessment, and HF phenotype, while LV volumes were more significantly reduced in HFrEF compared to HFpEF patients. A consonant pattern of effects on LVR was seen in all studies examined, with the exception of the REFORM trial [15], which was the only one showing an opposite direction of the effect of SGLT2i on LV volumes. This could be due to the fact that i both types of HF were analyzed in REFORM, without providing separate results.

Improvements in LVRR may be one of the proposed mechanisms by which SGLT2i can exert favorable effects on clinical outcomes. Indeed, in HF patients, drugs with positive effects on LV remodeling in the short term end up improving mortality in the long term [24]. Specifically, a sub-analysis of 30 mortality trials of 25 drug/device therapies and 88 remodeling trials of the same therapies, proved that the odds ratio for death in the mortality trials was significantly correlated with drug/device effects on LVEF (r = -0.51, p < 0.001), EDV (r = 0.44, p = 0.002), and ESV (r = 0.48, p = 0.002)^24^.

However, although reversal of LV remodeling is an important factor in reducing mortality and morbidity in HFrEF [25], it is unlikely that it could entirely explain all the beneficial prognostic effect observed with this class of drugs. Indeed, the SGLT2i-related outcome improvement reported in RCTs [1–4], as well as improvement in quality of life [26], appears very early after therapy is started, before a significant impact on LV volumes and shape could become appreciable.

The potential effect of SGLT2 inhibitors on LV structure and function is thought to be multifactorial and mediated predominantly by systemic hemodynamic and metabolic effects [27]. The failing heart is characterized by a myocardial metabolic remodeling [28], with a shift from free fatty acids (FFA) utilization of healthy myocardium, toward glucose consumption. From one hand, these adaptations lead to reduced myocardial oxygen requests, but they also decrease the production of adenosine triphosphate (ATP) molecules [29]. This latter effect tends to be compensated by oxidation of ketone bodies that are the most energetically efficient fuel because of the higher number of ATP molecules produced at the lowest oxygen requirements [29, 30]. It has been hypothesized that the positive effects of SGLT2i on LV structural remodeling might be mediated by their capability of improving myocardial energetics via switching myocardial metabolism from glucose to ketone bodies. Santos-Gallego [31] and coworkers, using a nondiabetic porcine model in which HF was induced after 2-hours balloon occlusion of the proximal left anterior descending artery, clearly demonstrated that, compared to control group, empagliflozin markedly improved LV remodeling indices (LV mass, dilatation, and LV sphericity) after 2 months. This effect was associated with a reduced uptake of myocardial glucose and less glucose-related enzymes in empagliflozin-treated pigs, suggesting a shift from glucose toward ketone bodies utilization, with consequent increase in myocardial ATP content and enhanced myocardial work efficiency [31]. This metabolic switch from the energy-inefficient glucose towards consumption of fatty acids and ketone bodies has also been confirmed in humans in HFpEF [32], and they are thought to mediate the improvement in LV remodeling after SGLT2i therapy. However, the recent EMPAVISION trial did not show improvement in myocardial energetics in patients with either HFrEF or HFpEF [33].

It is also possible that an improvement in cardiac microvascular endothelial cells could contribute the positive effects of SGLT2-inhibitors on LV remodeling, as evidenced by experimental data suggesting an enhanced bioavailability of endothelial-derived nitric oxide as a consequence of the capability of SGLT2 inhibitors to counteract the TNF-a-induced increase in the cytoplasmatic reactive oxygen species [34]. Other possible mechanisms of action of SGLT2-inhibitors are related to their capability to directly inhibit cardiac Na^+^/H^+^ exchanger (NHE) thus reducing Na^+^ concentrations in cardiomyocytes [35], which activity is generally increased in diabetic and failing hearts. Furthermore, SGLT2i ameliorate myocardial ischemia-reperfusion injury, reduce infarct size and microvascular obstruction, decrease apoptosis and oxidative stress [36].

The reduction of LVM index in HF patients observed in this meta-analysis represents another important effect of SGLT2i treatment, the mechanisms of which are not fully understood. It may either reflect a decrease in cardiomyocyte mass, or changes in interstitial water content, or both. It has also been supposed that the regression of LVM might be mediated by the hypotensive effect of SGLT2-inhibitors. However, in the EMPA-HEART trial the change in 24-hour ambulatory blood pressure was not related to the change in LV mass over 6-month [17] therapy, suggesting that the effect on LV mass could be at least partly unrelated to blood pressure reduction. Interestingly, in some studies the reduction in LV mass took place in the absence of concurrent reduction in LV volumes, thus reflecting an overall reduction in wall thickness [17]. Further studies focusing on detailed tissue characterization by CMR may contribute to understand which mechanisms is mostly responsible for the observed reduction in LVM. Finally, the dual SGLT1/2 inhbitor sotagliflozin is also being investigated in the SOTA-P-CARDIA trial (NCT05562063).

## Conclusions

Sodium-glucose cotransporter-2 inhibitors might play an interesting role in reversing adverse cardiac remodeling and improving LV systolic function in HF patients, mainly in those with HFrEF. Reversed cardiac remodeling may partially explain the favourable effects of SGLT2i on HF. These results should be interpreted in light of limitations including: (a) heterogeneity of the studies; (b) small sample size, in particular of the studies conducted with CMR; (c) different duration of the time to re-evaluation; (d) different types of SGLT2 inhibitors used.

### Electronic supplementary material

Below is the link to the electronic supplementary material.

Supplementary Material 1

## Data Availability

The data that support the findings of this study are available from the corresponding author upon reasonable request.
