# Supplementary material for: Left ventricular remodeling response to SGLT2 inhibitors in heart failure: an updated meta-analysis of randomized controlled studies
Source: Cardiovasc Diabetol. 2023 Sep 2;22:235. doi: 10.1186/s12933-023-01970-w (PMC10475184; doi:10.1186/s12933-023-01970-w)
Supplement: Supplementary file 1 — Supplementary Material 1 [file 12933_2023_1970_MOESM1_ESM.docx]

**SUPPLEMENTAL MATERIAL DATA**

## ***Term for searching***

(((((((((((((((((((((((((Sodium-Glucose Transporter 2 Inhibitors[Title/Abstract]) OR (SGLT2 Inhibitors[Title/Abstract])) OR (SGLT2 Inhibitors[Title/Abstract])) OR (SGLT-2 Inhibitors[Title/Abstract])) OR (SGLT 2 Inhibitors[Title/Abstract])) OR (empagliflozin[Title/Abstract])) OR (dapagliflozin[Title/Abstract])) OR (canagliflozin[Title/Abstract])) OR (ertugliflozin[Title/Abstract])) OR (tofogliflozin[Title/Abstract])) OR (luseogliflozin[Title/Abstract])) OR (sergliflozin[Title/Abstract])) OR (sotagliflozin[Title/Abstract])) OR (remogliflozin[Title/Abstract])) AND (cardiac function[Title/Abstract])) OR (systolic function[Title/Abstract])) OR (ventricular function[Title/Abstract])) OR (diastolic function[Title/Abstract])) OR (Diastole[Title/Abstract])) OR (ventricular volumes[Title/Abstract])) OR (left ventricular volume[Title/Abstract])) OR (ventricular remodeling[Title/Abstract])) OR (cardiac reverse remodeling[Title/Abstract])) OR (cardiac remodeling[Title/Abstract])) OR (left ventricular remodeling[Title/Abstract])) OR (ejection fraction[Title/Abstract])

***Filters applied***: Full text, Clinical Trial, Randomized Controlled Trial, human studies, English language, heart failure setting


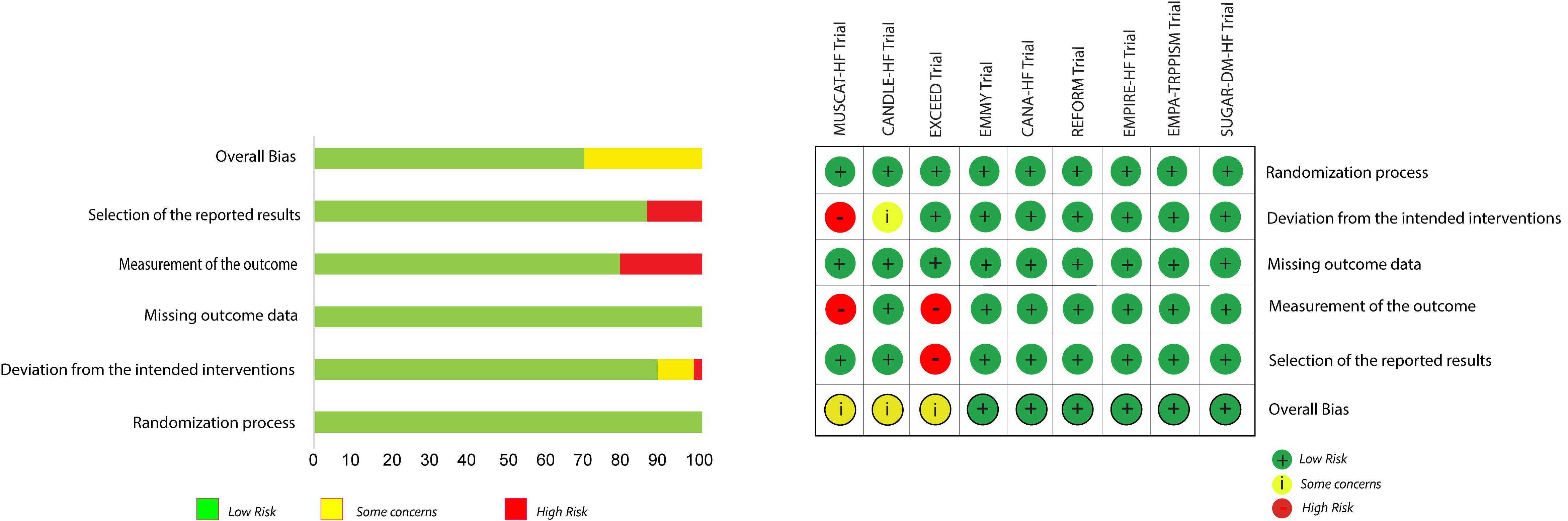


**Supplemental Figure S1**– Risk of bias graph (Left) and risk of bias summary for each included study (right).

| **Supplemental Table-S1: Risk of Bias assessment. The Begg adjusted-rank correlation test** | | | | | | |
| --- | --- | --- | --- | --- | --- | --- |
|  | ***Nonparametric rank correlation test (Begg)*** | | | **Regression-based Egger test** | | |
|  | **Kendall's score** | **SE** | ***P*** | ***Beta1*** | ***SE*** | ***P*** |
| End-Diastolic Volume | -8.00 | 9.59 | 0.4655 | 0.31 | 1.29 | 0.8121 |
| End-Systolic Volume | -10.00 | 9.59 | 0.3481 | -0.52 | 1.20 | 0.6673 |
| LV Ejection Fraction | 9.00 | 12.85 | 0.5334 | -0.44 | 1.17 | 0.7076 |
| LV Mass index | -10.00 | 9.59 | 3481 | -2.24 | 1.26 | 0.0774 |
| NT-proBNP | -5.00 | 5.32 | 0.4524 | -1.19 | 1.91 | 0.3161 |

**Selection bias**

All included studies were judged to have a low risk of selection bias.

**Performance bias**

Six studies were designed to be blinding of participants and personnel and were judged to have a low risk of performance bias. The other three studies, CANDLE, EXCEED, and MUSCAT-HF, were open-lable design so may confront high risk of performance bias.

**Attrition bias**

Eight studies were judged to have low risk of attrition bias. The CANDLE study were not based on intention-to-treat analysis and the sample size varied for each outcome variables, therefore may result in some risk of attrition bias.

**Reporting bias**

All included studies were rated as a low risk of reporting bias.


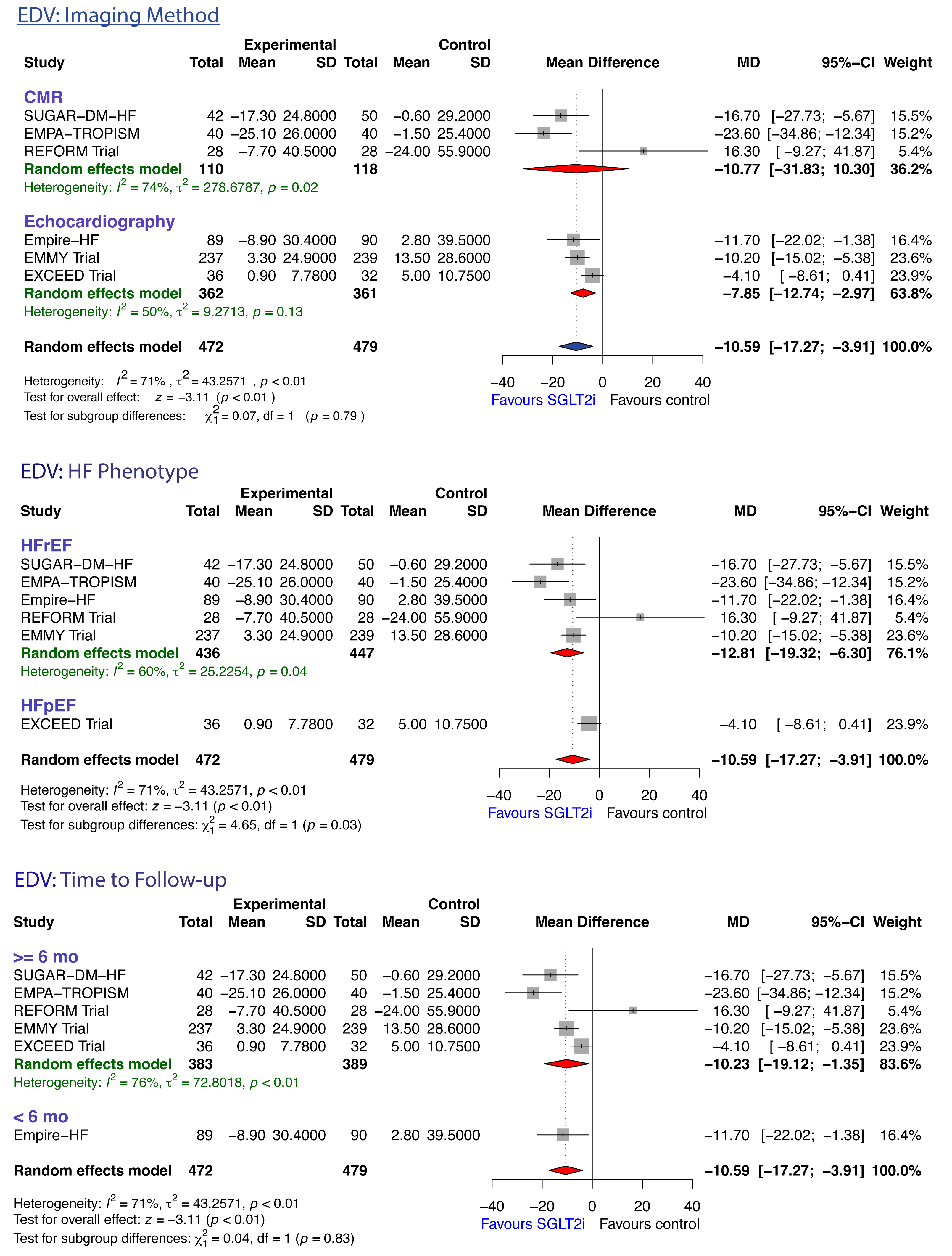


**Supplemental Figure-S2**: Forrest plot showing changes in end-diastolic volume from baseline to study endpoint in randomized controlled trials of heart failure patients treated with sodium glucose transporter-2 inhibitor therapy versus controls, according to pre-specified subgroups.


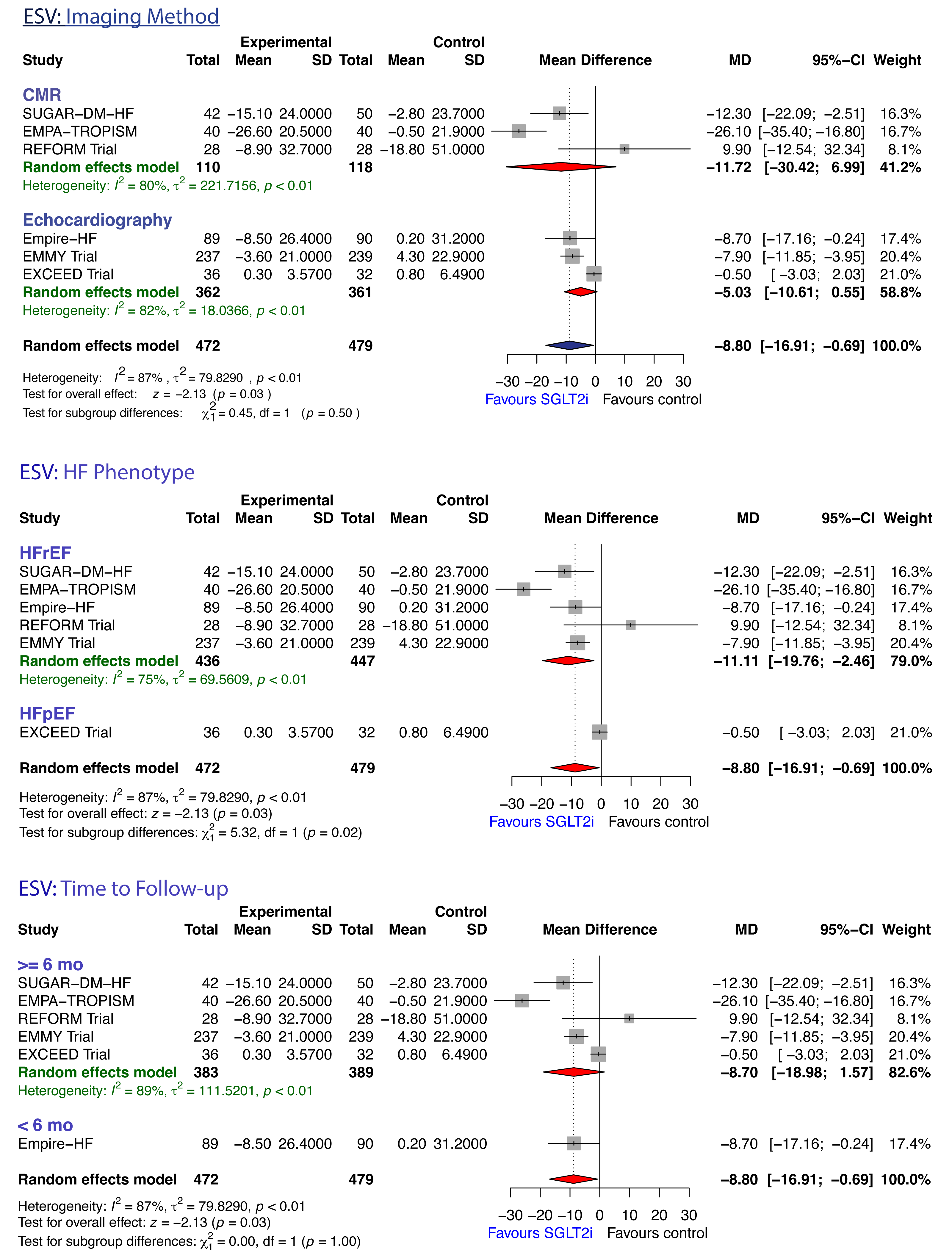


**Supplemental Figure-S3**: Forrest plot showing changes in end-systolic volume from baseline to study endpoint in randomized controlled trials of heart failure patients treated with sodium glucose transporter-2 inhibitor therapy versus controls, according to pre-specified subgroups.


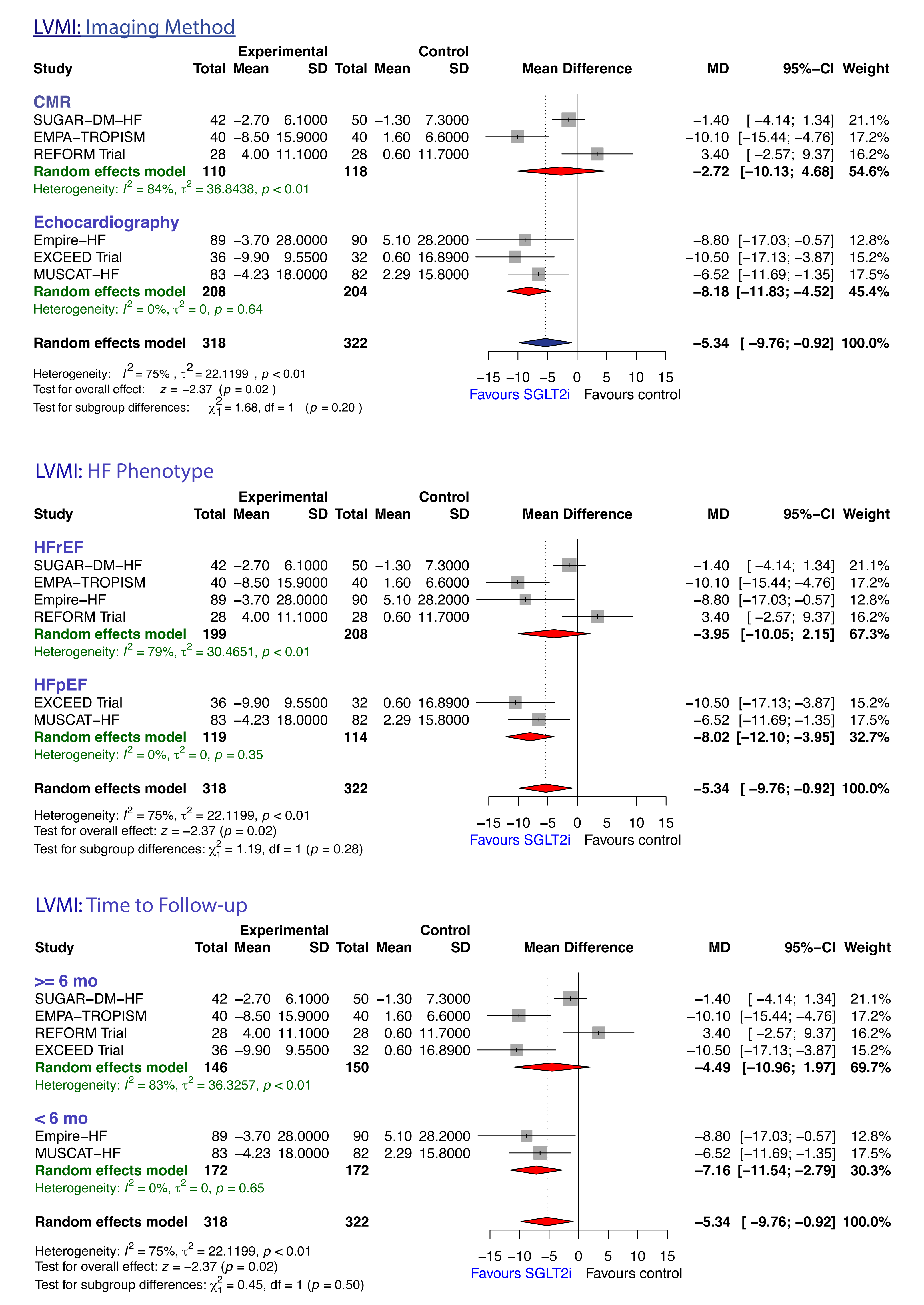


**Supplemental Figure-S4**: Forrest plot showing changes in LV Mass index from baseline to study endpoint in randomized controlled trials of heart failure patients treated with sodium glucose transporter-2 inhibitor therapy versus controls, according to pre-specified subgroups.


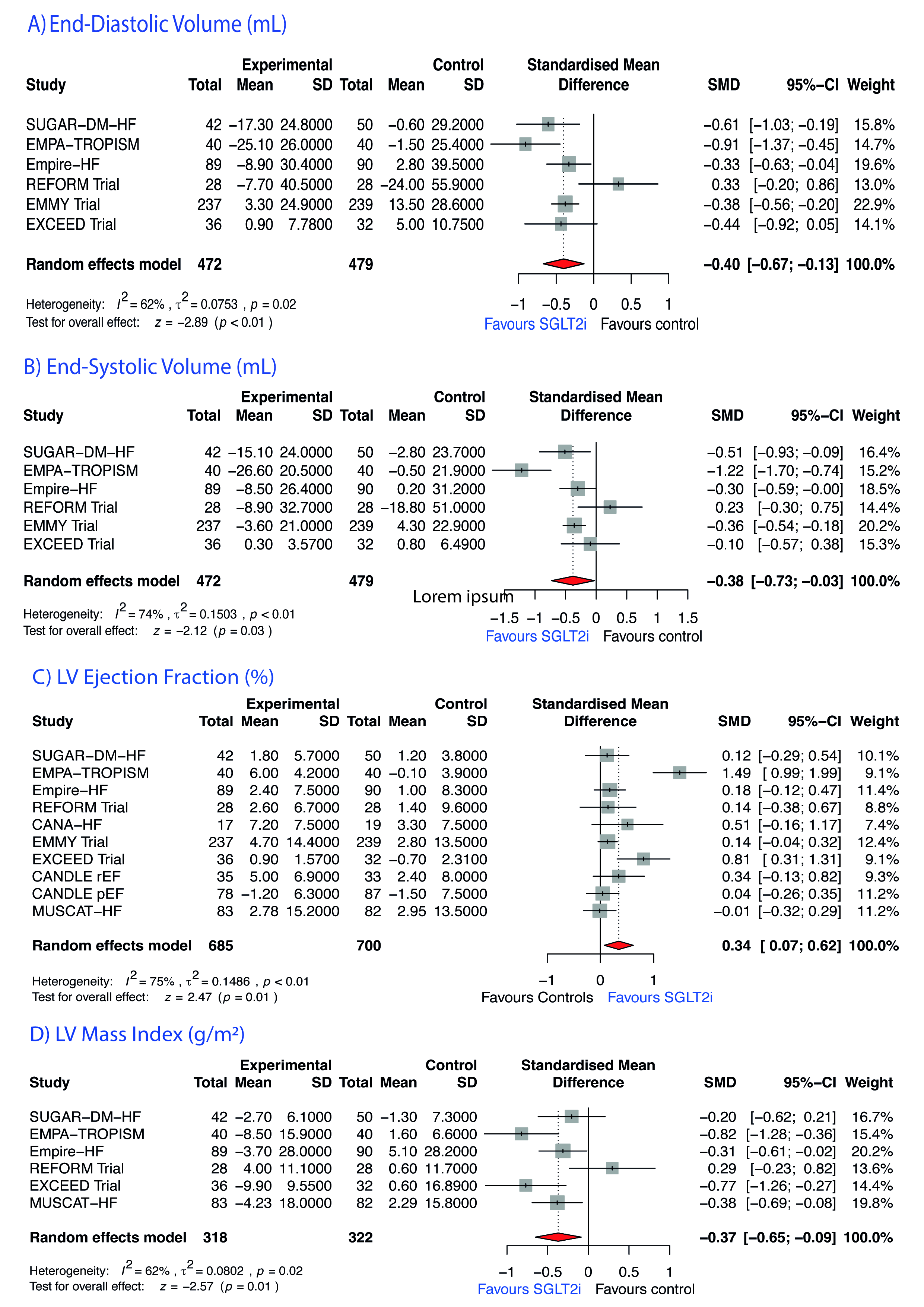


**Supplemental Figure-S5**: Forrest plot showing effect size as standardized mean difference (SMD) for changes in end-diastolic volume (top), end-systolic volume (bottom), LV Ejection Fraction, and LV Mass index from baseline to study endpoint in randomized controlled trials of heart failure patients treated with sodium glucose transporter-2 inhibitor therapy versus controls.
